# Expression Level of a Phenylalanine Ammonia-Lyase Gene in Poinsettia Is Negatively Correlated with Poinsettia Branch-Inducing Phytoplasma Titer

**DOI:** 10.1128/spectrum.03814-22

**Published:** 2022-11-29

**Authors:** Shin Lee, Chien-Young Chu, Chia-Ching Chu

**Affiliations:** a Department of Plant Pathology, National Chung Hsing University, Taichung, Taiwan; b Department of Horticulture, National Chung Hsing University, Taichung, Taiwan; c Advanced Plant Biotechnology Center, National Chung Hsing University, Taichung, Taiwan; USDA - San Joaquin Valley Agricultural Sciences Center

**Keywords:** phytoplasma concentration, population dynamics, gene expression, phenylalanine ammonia-lyase, infection density, poinsettia branch-inducing phytoplasma

## Abstract

Poinsettia is an important ornamental cultivated worldwide. Commercial poinsettias are almost universally infected with a pathogen known as the poinsettia branch-inducing phytoplasma (PoiBI), which can increase the level of branching in host plants and make the plants more desirable to consumers. Despite PoiBI’s crucial role in poinsettia production, little is known about PoiBI-poinsettia interactions in regard to the pathogen’s *in planta* population dynamics. The expression profiles of a phenylalanine ammonia-lyase gene (Euphorbia pulcherrima *PAL* [*EpPAL*]) and the PoiBI titers in poinsettia tissues were investigated. Differential gene expression analyses using quantitative PCR (qPCR) showed that *EpPAL* expression levels differed significantly across tissue types. The highest expression levels were detected in stems, followed by root. Lower *EpPAL* expression levels were detected in leaf tissues, particularly in source leaves closer to the base; the average expression level in these leaves was only one-seventh of that detected in stems. Phytoplasma concentrations in source leaves close to the base were significantly greater than the other tissue types; the average value was 7.6-fold of that detected in stem tissues, which had the lowest phytoplasma titers. A negative correlation between *EpPAL* expression level and PoiBI load was detected, suggesting that the products of *EpPAL*-associated pathways or other genes indirectly associated with *EpPAL* may interfere with PoiBI’s growth. While additional studies are needed to validate these interpretations, the results from this work provide new insights into PoiBI-poinsettia interaction and showed that correlations between pathogen load and defense-related genes could be detected in phytoplasma-associated pathosystems.

**IMPORTANCE** Phytoplasma-plant interactions are interesting subjects for fundamental and applicative research. Although many studies have characterized molecular interplays between these pathogens and hosts, knowledge on relationships between phytoplasmas’ *in planta* population dynamics and host gene expression remains scarce. By using the poinsettia branch-inducing phytoplasma (PoiBI) and poinsettia as a model system, a negative correlation was observed between the expression level of a plant defense-related gene and the pathogen’s titer. The findings provide potential explanations to PoiBI’s distribution patterns in the plant and highlight the importance of studying phytoplasma-plant interactions in regard to the pathogen’s population dynamics in other pathosystems.

## OBSERVATION

Poinsettia (Euphorbia pulcherrima) is one of the most important flowering ornamentals cultivated worldwide (1, 2). An interesting feature of commercial poinsettias is that almost all of them exhibit a free-branching phenotype attributed to the infection by a bacterium known as poinsettia branch-inducing phytoplasma (16SrIII-H; PoiBI) (2–4). Since free-branching poinsettias are ideal for developing potted flowers, PoiBI is introduced to newly bred cultivars, often by grafting, and maintained during the cutting propagation process (2). Distribution of PoiBI is uneven within poinsettia plants; PoiBI loads tend to be greater in lower, unfolded leaves (5) than in other plant parts. Previous reports also showed that greater PoiBI loads in stock plants increase the likelihood of them producing new plants with higher levels of branching (5). Despite PoiBI’s significance in poinsettia’s commercial value, little has been explored regarding factors linked to PoiBI’s population dynamics.

Phenylalanine ammonia-lyases (PALs) are enzymes associated with the phenyl-propanoid pathway in plants, which is responsible for the production of compounds like anthocyanins, lignin, flavonoids, polyphenols, and salicylic acid (6–8). As such, PALs and the expression of their genes (*PAL*) can play crucial roles in plant development (9) and contribute to the plants’ defense against microbial pathogens (6). Studies on other plant systems reported induction in PAL activity or *PAL* expression resulting from phytoplasma infections (10, 11). Research also suggested that polyphenols produced by PAL-associated pathways may contribute to defense against phytoplasmas (12). Thus, it is possible that *PAL* expression in poinsettia may be associated with *in planta* titers of PoiBI. To test this hypothesis, a *PAL* gene that has been cloned and studied in a previous work (13) was examined for its expression level’s association with PoiBI load using quantitative PCR (qPCR).

**FIG 1:**
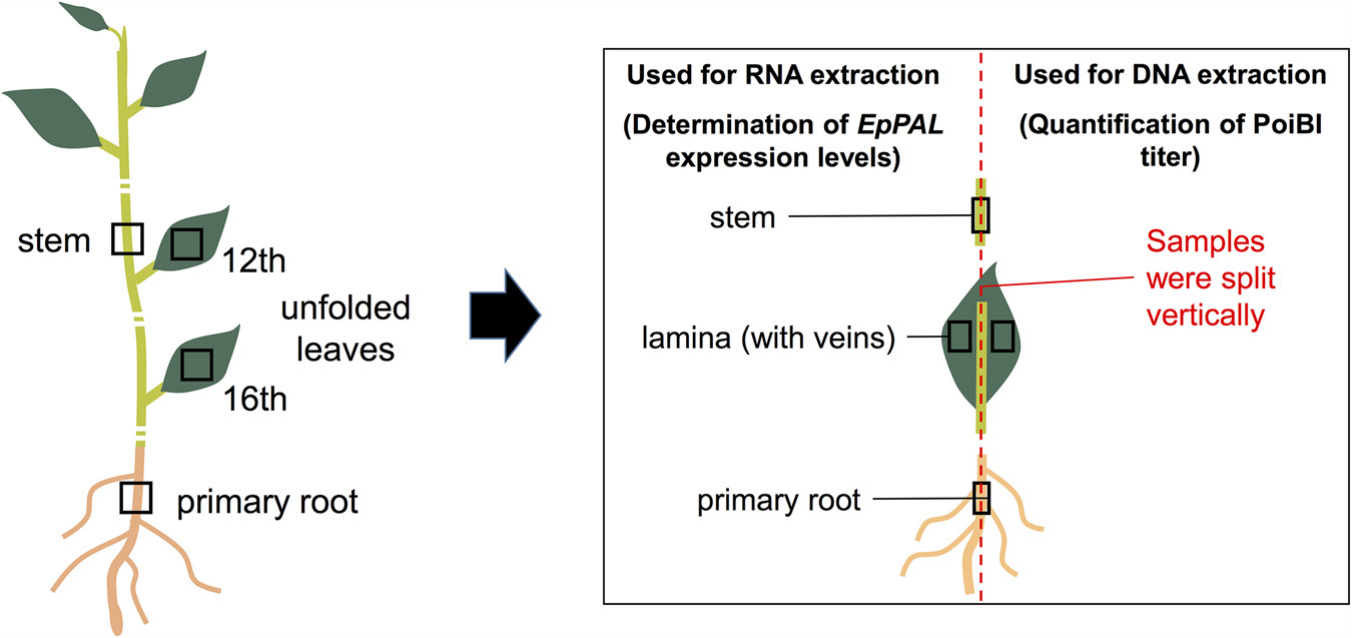
Six PoiBI-infected poinsettias were used in this study. The plant parts sampled included lamina tissues (including veins) of the 12th and 16th unfolded leaves (counting from the top), stem tissues just above the 12th unfolded leaves, and primary root tissues. Every sample was split vertically; one half of each sample was used for RNA extraction and gene expression analyses, while the other half was used for DNA extraction and quantification of PoiBI titer.

Vegetative-stage poinsettia ‘Luv U Pink’ plants were used throughout this study; a previous work showed that the cultivar carries only PoiBI (5). Six plants were cutting propagated at the same time and grown for 85 days. Thereafter, four types of plant tissues were sampled from each plant; these included lamina tissues (including veins) of the 12th and 16th unfolded leaves (counting from the top), stem tissues just above the 12th unfolded leaves, and the primary root tissues (Fig. 1). During sampling, each sample was split vertically; one half of the sample was used for DNA extraction and the other half was used for RNA extraction and generation of cDNA samples (see the supplemental material).

The expression levels of *E. pulcherrima* PAL (*EpPAL*) in different plant tissues were quantified by conducting qPCR assays on the cDNA samples. For these tests, primer pairs specific to poinsettia’s *EpPAL* and elongation factor 1α gene (*EpEF*; housekeeping gene; internal reference) (14) were used, and the data were analyzed with the comparative threshold cycle (delta-delta-*C_T_*) method (15) (supplemental material). The titers of PoiBI (phytoplasma concentrations) were determined through qPCRs coupled with plasmid-based standard curves; these tests were conducted on poinsettia DNA samples as described previously (5). The assays quantified the copy numbers of PoiBI’s 16S rRNA gene in the samples using phytoplasma-specific primers and probe (see Table S1 in the supplemental material) designed in a previous work (16). Because phytoplasmas have two copies of the 16S rRNA gene in their genomes (17), the copy number of each sample was first divided by 2; the resulting value was then normalized (divided) by that of the poinsettia gene *EpPAL* (supplemental material) (18, 19).

**FIG 2:**
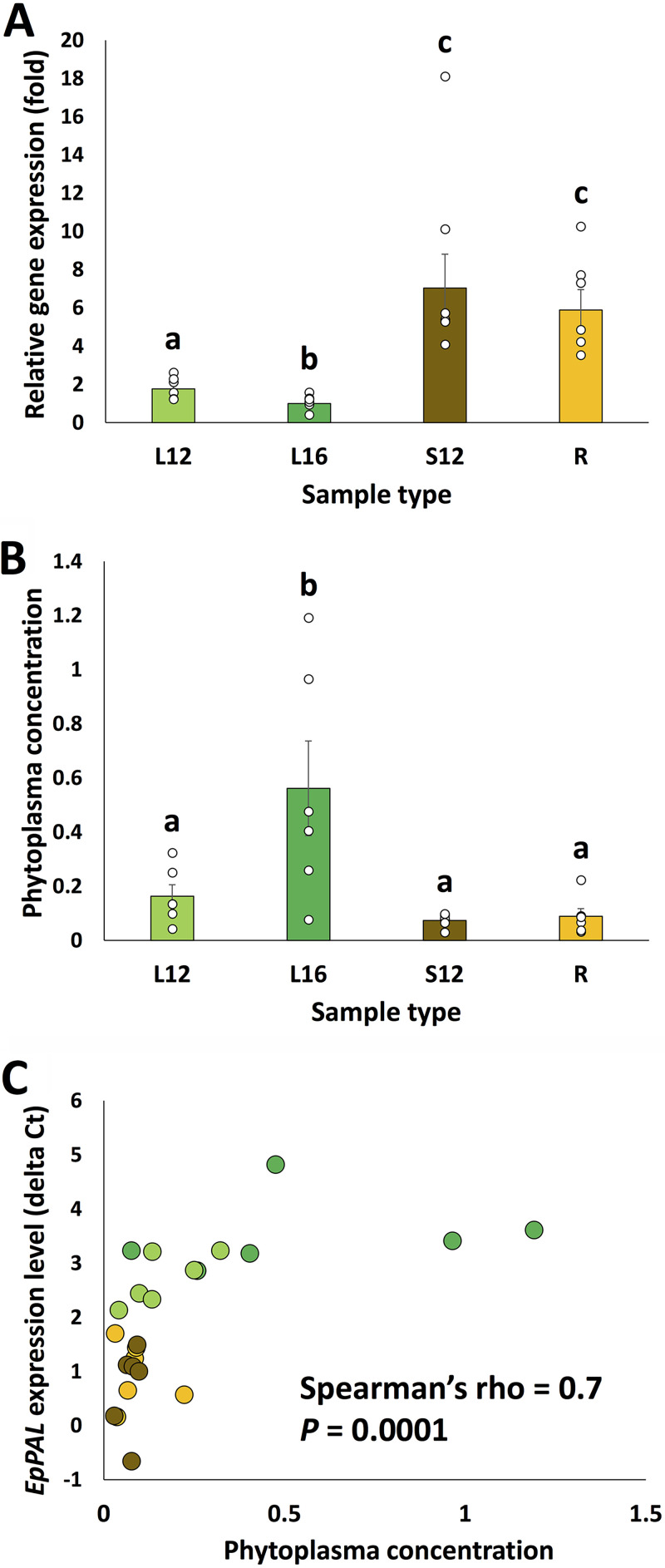
(A and B) Phenylalanine ammonia-lyase gene (*EpPAL*) expression levels (A) and phytoplasma concentrations (B) in different tissue types of poinsettias. For the gene expression analysis, the elongation factor 1α gene (*EpEF*) was used as a reference gene and the data were analyzed using the delta-delta-*C_T_* method. Each sample’s phytoplasma concentration was calculated by dividing the 16S rRNA gene’s copy number by that of *EpPAL*. Different letters above the bars represent significant differences between groups (Fisher’s LSD; *P* < 0.05), and the error bars indicate standard errors. L12, the 12th unfolded leaves (counting from the top); L16, the 16th unfolded leaves; S12, stems located above L12; R, primary roots. (C) Scatterplot showing a negative correlation between *EpPAL* expression level and phytoplasma concentration. Gene expression levels were represented as delta-*C_T_* values; higher values indicate lower expression levels. The data points are color coded based on their sample types as in panels A and B.

After obtaining the results from the differential gene expression analysis, a two-way analysis of variance (ANOVA) (tissue type × plant difference [random factor]) without interaction was conducted on the delta-*C_T_* values, and both factors were found to significantly influence *EpPAL* expression (tissue type, *F* = 40.62, *P* < 0.0001; plant difference, *F* = 3.1, *P* = 0.04). Further *post hoc* testing revealed that *EpPAL* expression levels were significantly higher in the stems and primary roots than in the unfolded leaves; also, the expression levels in the 12th unfolded leaves were significantly higher than those in the 16th unfolded leaves (Fig. 2A; Fisher’s least significant difference [LSD]; *P* < 0.05). The average expression level in stem tissues was 7-fold that detected in the 16th unfolded leaves. The pattern is generally consistent with those of an earlier study on PoiBI-infected poinsettia (13), except that the expression levels in stem tissues were relatively low in that study and higher in this work (Fig. 2A). Since the exact stem segments sampled were not specified in the previous study (13), it is likely that the distinct *EpPAL* expression patterns could be caused by sampling variations. It is also possible that the different results were due to variation between poinsettia variety; the previous study used the variety ‘Early Velvet’ (instead of ‘Luv U Pink’) for the assays (13).

A two-way analysis using the same design (tissue type × plant difference [random factor]) was also conducted on the PoiBI concentration values, and the results showed that tissue type was a significant factor associated with PoiBI load (*F* = 6.76, *P* = 0.004), while plant difference was not (*F* = 1.26, *P* = 0.33). The PoiBI concentrations tended to be higher in the leaf samples; PoiBI loads in the 16th unfolded leaves were significantly higher than those detected in the other sample types (Fisher’s LSD; *P* < 0.05; Fig. 2B). Average phytoplasma concentrations in the 16th unfolded leaves were 7.6-fold the values detected in stem tissues. Such a pattern is consistent with findings from a previous study (5), in which it was hypothesized that photosynthates are more abundant in the source leaves and that they could facilitate phytoplasma growth (5). Interestingly, a Spearman rank-order correlation test showed that PoiBI load is negatively correlated with *EpPAL* expression level (Spearman’s rho = 0.7; *P* = 0.0001) (Fig. 2C). Past studies have further suggested that polyphenols may contribute to host defense against phytoplasmas (12). Based on these facts, the patterns observed in this work could indicate that higher *EpPAL* expression facilitates poinsettia’s defense against pathogens, thereby interfering with PoiBI multiplication. It is also possible that there are other genes and gene products whose expression and functioning are correlated with *EpPAL* expression level and that they are the main cause of the pathogen’s distribution pattern. Although further testing is needed to determine the exact mechanisms underlying this pattern and to validate the proposed interpretations of the results, the data presented here clearly showed that correlations between pathogen load and defense-related genes could be detected in phytoplasma-associated disease systems. The observation highlights the importance of studying phytoplasma-plant interactions while considering the pathogen’s population dynamics.
