# Supplementary material for: Expression Level of a Phenylalanine Ammonia-Lyase Gene in Poinsettia Is Negatively Correlated with Poinsettia Branch-Inducing Phytoplasma Titer
Source: Microbiol Spectr. 2022 Nov 29;10(6):e03814-22. doi: 10.1128/spectrum.03814-22 (PMC9769536; doi:10.1128/spectrum.03814-22)
Supplement: Supplemental file 1 — Supplemental methods and Table S1. Download spectrum.03814-22-s0001.pdf, PDF file, 0.2 MB [file spectrum.03814-22-s0001.pdf]

SUPPLEMENTARY MATERIAL

MATERIALS AND METHODS

Cutting propagation. Young shoots with seven unfolded leaves were obtained from poinsettia ‘Luv U Pink’. The bases of the cuttings were dipped in rooting powder (2 g/kg 1-Naphthaleneacetic acid) and inserted into rooting cubes. After growing 15 d under a mist system, rooted plants were moved to 5-inch pots, and grown for another 70 d in a nursery (29°C day/22°C night) until sampling.

Sampling and storage of poinsettia tissues. Samples used for RNA extraction were placed in microcentrifuge tubes containing RNA*later* (Thermo Fisher Scientific), while those used for DNA extraction were placed separately in ziplock bags; all samples were kept at -80°C until use.

RNA and DNA extraction. Plant RNA was extracted using an E.Z.N.A. Plant RNA Kit (Omega Bio-Tek) and a TissueLyser II homogenizer (Qiagen). For each sample, approximately 0.05 g of tissue was used as the starting material. Extracted samples were examined using NanoDrop One spectrophotometer (Thermo Fisher Scientific). For reverse transcription, each cDNA sample was synthesized using 250 ng of RNA and the SuperScript IV VILO Master Mix (Thermo Fisher Scientific). The cDNA samples were 10-fold diluted and used for differential gene expression analyses.

Plant DNA was extracted with a Synergy 2.0 Plant DNA Extraction Kit (OPS Diagnostics) and a TissueLyser II. For each DNA sample, the amount of poinsettia tissue used was 0.02-0.05 g. The concentrations of the DNA samples were measured using a

NanoDrop One. The samples were stored at -20°C until use.

Generation of plasmid-based standard curves for qPCR assays. Prior to the qPCR tests, the target amplicons were amplified with conventional PCR. For both *EpPAL* and *EpEF*, the assays were conducted with the GoTaq Green Master Mix (Promega). Each 20-µl reaction included 0.4 µM of each primer (Table S1) and 1 µl of 10-fold diluted cDNA. The cycling conditions were 2 min at 95°C, 35 cycles of 30 s at 95°C, 30 s at 60°C, 1 min at 72°C, followed by 10 min at 72°C. The amplicons were cloned with the pGEM-T Easy Vector System (Promega). Plasmids carrying the target gene fragments were extracted from *Escherichia coli* JM109 transformants using a QIAprep Spin Miniprep Kit (Qiagen) and digested with PstI-HF (New England Biolabs). Linearized plasmids were recovered using QIAquick Gel Extraction Kit (Qiagen), quantified using NanoDrop One and diluted. Whenever qPCR assays were conducted, the plasmids were serial-diluted and tested along with the plant DNA samples. To assure that the qPCR results were comparable among different runs, the standard curves for each gene in each set of data were constructed using aliquots of the same preparation of linearized plasmids.

Differential gene expression assays. For the gene expression assays, all qPCR runs were completed using the iQ SYBR Green Supermix and a CFX Connect Real-Time PCR Detection System (Bio-Rad). Quantification of *EpPAL* copy numbers was achieved using primers PAL1F and PAL1R (Table S1) designed in a previous study (1). The assays were conducted in 20-µl reactions including 0.4 µM of each primer (Table S1) and 1 µl of cDNA. The qPCR conditions were set as previously described (1). For *EpEF*, primers EpEFfwd and EpEFrev (Table S1) developed in a previous work (2) was used. The 20-µl qPCR reactions for these tests contained 0.25 µM of each primer and 1 µl of cDNA. The qPCR conditions

were 3 min at 95°C, 40 cycles of 10 s at 95°C, 10 s at 58°C, 30 s at 72°C, followed by a dissociation curve assay. Each cDNA and plasmid DNA sample had three technical replicates and all assays included no-template controls. Differential gene expression analysis was conducted using the delta-delta-Ct method (3) and delta-Ct values were analyzed with statistical tests.

Quantification of phytoplasma concentrations. Phytoplasma concentrations were calculated based on data generated from qPCR tests coupled with plasmid-based standard curves.

The 16S rRNA gene's copy numbers were determined with TaqMan assays using primer set CYS2Fw/CYS2Rv and the probe CYS2Probe (Table S1) designed in a previous study (4). SYBR green assays using primers PAL1F and PAL1R (Table S1) were conducted to determine *EpPAL*'s copy numbers. Similar to the gene expression analyses described above, PCR was conducted prior to the qPCR assays, and the target amplicons of both genes were cloned; assays using primer pair CYS2Fw/CYS2Rv and poinsettia DNA were conducted as previously described (1); assays using PAL1F and PAL1R were conducted as described above (using plant DNA instead of cDNA as templates). After cloning the amplicons, the plasmids were extracted, linearized, purified, and diluted to 5 ng/µl. Serial-diluted plasmids were tested along with the plant DNA samples, allowing absolute quantification of the genes' copy numbers (5).

The amplification conditions for the qPCR tests were set as previously described (1), and 10 ng of template DNA was used for each reaction. The CFX Connect Real-Time PCR Detection System (Bio-Rad) was again used for these assays. The TaqMan assays were completed using the iTaq Universal Probes Supermix (Bio-Rad), while the SYBR green tests were conducted with the iQ SYBR Green Supermix (Bio-Rad). Each plant DNA and plasmid

DNA sample had three technical replicates and all runs included no-template controls. The copy numbers of the two genes in the tested samples were calculated based on the standard curves as previously described (5, 6).

Because previous studies reported that phytoplasmas have two copies of 16S rRNA gene in their genomes (7), the 16S rRNA gene's copy number of each sample was first divided by 2; the resulting value was then normalized (divided) by *EpPAL*'s copy number.

Data analysis. Only data generated from qPCR runs with PCR efficiencies above 87.2% ($R^2 > 0.99$) were used for statistical analysis. Two-way analysis of variance [ANOVA; "tissue type" x "plant difference" (random factor)] tests without interaction were conducted on *EpPAL* expression (delta-Ct) and PoiBI titer (phytoplasma concentration) values. Post-hoc tests were conducted with the Fisher's least significant difference (LSD) procedure. The association between *EpPAL* expression levels (delta-Ct) and PoiBI titers were examined using a Spearman's rank-order correlation test.

SUPPLEMENTARY TABLE

Table S1. Primers and probes used in this study.

Target gene	Assay	Primer/Probe sequences (5' to 3')	Annealing temperature	Reference
Poinsettia's phenylalanine ammonia-lyase gene (*EpPAL*)	SYBR green qPCR assay	PAL1F: CTAGCAGCGATTGGGTTATG<br>PAL1R: ACCCTGCTTTGTTCTTCTATG	60°C	(1)
Poinsettia's elongation factor 1α gene (*EpEF*)	SYBR green qPCR assay	EpEFfwd: GCTTGGAGGTATTGACAAGCGTG<br>EpEFrev: ATGATGAGAACAGCACAGTCAGC	58°C	(2)
Phytoplasma's 16S rRNA gene	TaqMan qPCR assay	CYS2Fw: AGGTTGAACGGCCACATTG<br>CYS2Rv: TTGCTCGGTCAGAGTTTCCTC<br>CYS2Probe: ACACGGCCCAAACTCCTACGGGA	60°C	(4)

REFERENCES

1. Lee S, Chu C-Y, Chu C-C. 2021. Variability of phytoplasma infection density in poinsettia and evaluation of its association with the level of branching in host plants. Plant Dis 105:1539-1545.
2. Nicolaisen M, Horvath DP. 2008. A branch-inducing phytoplasma in *Euphorbia pulcherrima* is associated with changes in expression of host genes. Phytopathology 156(7-8):403-407.
3. Livak KJ, Schmittgen TD. 2001. Analysis of relative gene expression data using real-time quantitative PCR and the 2[-Delta Delta C(t)] method. Methods 25:402-408.
4. Galetto L, Bosco D, Marzachì C. 2005. Universal and group-specific real-time PCR diagnosis of flavescence dorée (16Sr-V), bois noir (16Sr-XII) and apple pro- liferation (16Sr-X) phytoplasmas from field-collected plant hosts and insect vectors. Ann Appl Biol 147:191-201.
5. Whelan JA, Russell NB, Whelan MA. 2003. A method for the absolute quantification of cDNA using real-time PCR. J Immunol Methods 278:261-269.
6. Ikten C, Ustun R, Catal M, Yol E, Uzun B. 2016. Multiplex real-time qpcr assay for simultaneous and sensitive detection of phytoplasmas in sesame plants and insect vectors. PLoS ONE 11(5):e0155891.
7. Schneider B, Seemüller E. 1994. Presence of two sets of ribosomal genes in phytopathogenic mollicutes. Appl Environ Microbiol 60:3409-3412.
